# New Biopharmaceutical Characteristics of In Situ Systems Based on Poloxamer 407

**DOI:** 10.3390/gels9070508

**Published:** 2023-06-21

**Authors:** Elena O. Bakhrushina, Elizaveta V. Novozhilova, Marina M. Shumkova, Victor S. Pyzhov, Maria S. Nikonenko, Alexander I. Bardakov, Natalia B. Demina, Ivan I. Krasnyuk, Ivan I. Krasnyuk

**Affiliations:** 1 Department of Pharmaceutical Technology, A.P. Nelyubin Institute of Pharmacy, I.M. Sechenov First Moscow State Medical University (Sechenov University), Moscow 119048, Russia; bakhrushina_e_o@staff.sechenov.ru (E.O.B.); elizaveta.novozhilova@stud.unifi.it (E.V.N.); pyzhov_v_s@student.sechenov.ru (V.S.P.); nikonenko_m_s@student.sechenov.ru (M.S.N.); bardakov_a_i@staff.sechenov.ru (A.I.B.); demina_n_b@staff.sechenov.ru (N.B.D.); krasnyuk_i_i@staff.sechenov.ru (I.I.K.J.); 2 Department of Chemistry “Ugo Schiff”, Università di Firenze, Via Della Lastruccia 3-13, 50019 Sesto Fiorentino, Italy; 3 Department of Analytical, Physical and Colloidal Chemistry A.P. Nelyubin Institute of Pharmacy, I.M. Sechenov First Moscow State Medical University (Sechenov University), Moscow 119048, Russia; krasnyuk_i_i_1@staff.sechenov.ru

**Keywords:** in situ systems, Kolliphor, poloxamer, polyethylene glycol, gelation temperature, adhesion

## Abstract

Thermosensitive systems based on poloxamer 407 are widely used in targeted drug delivery; however, the stability of the phase transition temperature remains insufficiently studied. This article presents the results of a study on the effect of adding polyethylene glycols (PEG) with different molecular weights and some classical gel-forming polymers on the gelation temperature of thermoreversible compositions based on poloxamer 407 in a long-term experiment. The study showed a positive effect of PEG addition with average molecular weights at concentrations of 1.5–2.0%, as well as gelling agents at a concentration below the critical gelation concentration. The proposed rheological test for studying the samples’ adhesion can give an indirect forecast of the composition adhesive rate. Based on the conducted studies, three experimental binary systems based on poloxamer 407 were selected, with the addition of HPMC 0.5%, sodium alginate 0.5%, and PEG 1500 1.5%. These systems are the most promising for the further development of in situ targeted drug delivery systems.

## 1. Introduction

Thermosensitive in situ systems based on poloxamers have become one of the most popular products for targeted therapy in the last decade. Based on thermosensitive in situ systems, drugs for parenteral, intranasal, vaginal, rectal, and ophthalmological usage are being developed [1,2,3,4].

In comparison to many other directionally synthesized polymers, poloxamers are commercially available, the technology of obtaining targeted delivery systems based on them is highly cost-effective, and patients’ medical adherence to therapy with their use is high. In addition to this, poloxamers, undergoing sol–gel conversion and back, do not change their rheological and technological characteristics [5].

Despite the significant advantages, monocomponent aqueous solutions of poloxamers have several disadvantages: a relatively low gelation temperature (26–27 °C for poloxamer 407), average adhesive ability, and low adsorption activity [6,7,8]. Due to the narrow range of concentrations used to produce thermoreversible dosage forms (18.0–20.0%), it is possible to vary the technological characteristics of the finished dosage form by introducing an additional polymer into the composition. The resulting binary or non-binary polycomplexes could either improve thermoreversible properties or result in their loss [9].

At the moment, many studies have proven the feasibility of creating polycomplexes based on poloxamers, since these binary and non-binary systems not only have improved technological characteristics and are more relevant to the physiological conditions of the sol–gel transition, but also have improved biopharmaceutical characteristics [7,10,11,12,13]. Thus, Ricci et al. in their studies demonstrated that the introduction of polyethylene glycol 400 or inorganic salts into a poloxamer solution causes an increase in the active pharmaceutical ingredient (API) release and diffusion coefficients [14,15]. In Wang P. et al., the effect of the multi-gel core of poloxamer 407 with polyethylene glycol on the completeness of the Exenatide release profile from microspheres was shown [16].

The standard methods of investigation for in situ systems based on poloxamers are the determination of the gelation temperature, gelation time, and gel strength [17,18,19]. Additionally, adhesive (bioadhesive) properties of pharmaceutical compositions are often studied, and structural and mechanical analyses are performed on a viscometer and a texture analyzer [18,20,21].

Rheological analysis is an in vitro method for determining adhesion, which makes it possible to predict the behavior of polymer matrices in vivo, as well as to investigate their structural interactions by interpreting changes following physicochemical interactions [22]. The mucoadhesion process is a phenomenon that combines different types of interactions, and a rheological method is used to evaluate these interactions between mucin and a gel-forming polymer [23,24].

For the first time, the use of rheological indicators to determine the degree of adhesion to the mucosa was proposed by Hassan and Gallo in their 1990 paper [25]. Today, some studies demonstrate a correlation between the adhesion parameters of polymer matrices measured using classical methods (such as the flow method, separation force from the mucin layer, and the use of texture analyzers) and rheological measurements of polymer matrices at low shear rates (up to 20 s^−1^) [26].

The range of shear rates chosen for the developed technique was from 0 to 10 s^−1^. In vivo, the forces counteracting adhesion are influenced by gravity and the slope of the drug application surface. Consequently, the shear rate magnitude formed during the flow and separation of the adhesive sample from the mucous membrane is relatively small. 

In the study conducted by Bakhrushina et al., the potential of utilizing the rheological method without the addition of a mucin solution was demonstrated. This approach allows the obtention of primary data suitable for the screening stages of promising formulations. The experiment involved a diverse range of known polymers, and formulations both with and without the addition of mucin exhibited similar characteristics in terms of the nature of the rheological curve and shear stresses within the measured range [27].

Thus, it was concluded that the screening of samples can be carried out through a general visual assessment of the yield curve type, including its shape, smoothness, peaks, plateau zone, and shear stress range.

At the same time, the gelation temperature parameter, which is the main screening parameter when choosing the pool of optimal compositions, is not studied by most researchers of dynamics and, as a rule, is tracked only for several weeks from the moment of sample production. At the same time, the loss of the initial gelation temperature due to thermosensitive complexes is the main problem that prevents the widespread introduction of thermosensitive in situ systems into clinical practice. Stability is crucial in thermosensitive systems for targeted drug delivery during storage as well as after administration, as it can protect the encapsulated drugs from degradation and dilution upon administration. It also improves the expected treatment effect by releasing a specific load and controlling the systemic distribution of therapeutic drugs [28]. Studies by Grela et al. [20] show that in monocomponent placebo formulations based on poloxamer 407, significant changes in the main rheological parameters are observed that exceed the established criteria (±10% of each initial value). In addition, a complete loss of thermal reversibility was observed in samples stored at 40 °C.

In the study by Ivanova et al. [29], the problem of the stability of monocomponent compositions based on poloxamer 407 is proposed to be solved by adding various glycols, including propylene glycol and polyethylene glycol. The positive effect of glycols of different molecular weights on the stability of the liquid crystal gel phases of the poloxamer solution was established. Thus, the creation of poloxamer 407 polycomplexes with various excipients may also be advisable to increase the stability of the thermosensitive system.

This study aimed to investigate various polycomponent complexes containing poloxamer 407 for a determination of the stability of the gelation temperature in a long-term experiment, as well as the use of a rheological method to study the main parameters of thermosensitive in situ systems.

## 2. Results and Discussion

### 2.1. Results

#### 2.1.1. Gelation Temperature Screening Test

After preparation, all experimental samples were stored for 7 days for structuring. The structuring of the samples was necessary because, during the first 7 days, the temperature of the sol–gel transition of copolymers with PEGs was above 45 °C. Then, it rapidly decreased and reached a plateau by the seventh day. Because of this, the temperature values of the first 7 days were not taken into account since many samples subsequently showed stability.

**Figure 1:**
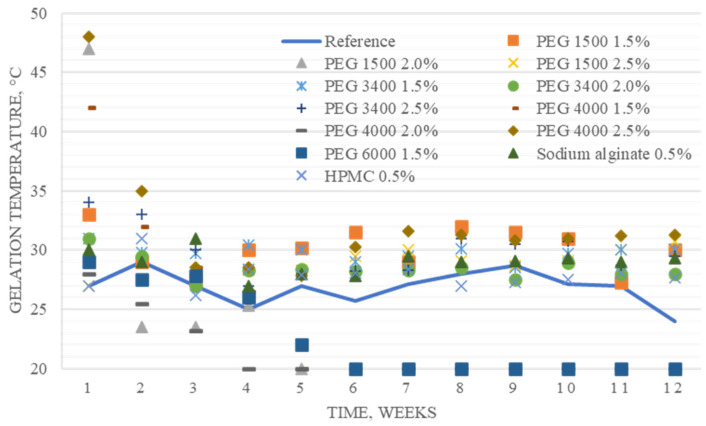
Dynamics of the gelation temperature change over 12 weeks for experimental samples.

After that, samples with aggregate stability were selected for further study, showing no signs of delamination, coagulation, or syneresis. All the selected compositions were transparent homogeneous liquids at the storage temperature. As a result of the initial selection, 12 experimental samples were selected for the experiment on long-term storage and the study of gelation temperature stability, in addition to the thermoreversible component (poloxamer 407, 18.0%) containing additional gelling agents in the following concentrations: PEG 400 1.5%; PEG 1500 1.5%, 1.0%, 2.5%; PEG 3400 1.5%, 2.0%, 2.5%; PEG 4000 1.5%, 2.0% 2.5%; PEG 6000 1.5%; HPMC 0.5%; sodium alginate 0.5%. A monocomponent composition of poloxamer 407 in a concentration of 18.0% and water was used as a reference sample. Figure 1 shows the dynamics of change in the analyzed indicator observed over 12 weeks. 

Statistical analysis was used to determine the statistical significance of differences in gelation temperatures and the gelation time of samples (Table 1). It was shown that the introduction of additional gel-forming agents (PEG 1500, PEG 3400, sodium alginate, and HPMC) not only increased the average gelation temperature but also made this indicator stable during the experiment.

The gelation time of the studied samples was not exposed to significant fluctuations during the entire storage period and varied in the range from 40 s (poloxamer 407 18.0%, PEG 4000 2.0%) to 1 min 51 s on average (poloxamer 407 18.0%, PEG 4000 2.5%).

#### 2.1.2. Rheological Properties of Selected Samples

**Figure 2:**
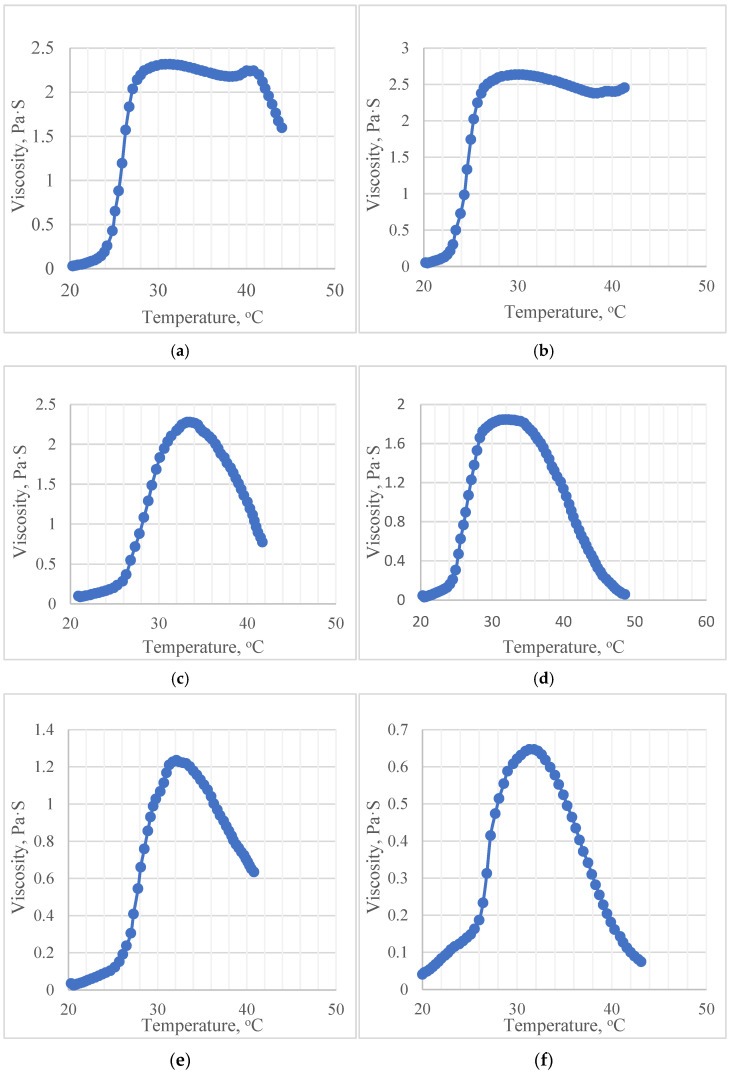
Rheograms of the studied samples: (**a**) poloxamer 407 18.0%; (**b**) poloxamer 407 18.0%, HPMC 0.5%; (**c**) poloxamer 407 18.0%, sodium alginate 0.5%; (**d**) poloxamer 407 18.0%, PEG 1500 1.5%; (**e**) poloxamer 407 18.0%, PEG 1500 2.5%; (**f**) poloxamer 407 18.0%, PEG 3400 1.5%.

At the next stage, the characteristics of the five selected stable samples were investigated in comparison with the reference composition using a rotational viscosity test. Figure 2 shows rheograms based on the results of measurements in the temperature range from 20 to 50 °C. All rheograms graphically confirm the presence of thermoreversible gel formation in both the reference and five multicomponent compositions. On the rheograms, a growth zone, a decline zone, as well as a plateau zone of dynamic viscosity (measured at a shear rate of 100 s^−1^) are distinguished.

The longest plateau zone was observed in the reference sample (Figure 2a), as well as in the sample containing HPMC in a concentration of 0.5% as an additional gel-forming agent (Figure 2b). At the same time, it should be noted that the stable high dynamic viscosity (2.5 ± 1.0 Pa·S) of the sample with HPMC was maintained at a temperature of 40 °C. Thus, the temperature of the reverse gel–sol transition for the studied complex was significantly higher than that for the other samples.

In the sample with alginate (Figure 2c), a stable high viscosity was observed at a temperature of 32 °C (2.17 Pa·S) but at a temperature of 35 °C, the value of the dynamic viscosity began to decrease, which indicates an opposite gel–sol transition in this temperature range. Thus, the plateau zone of a sample containing sodium alginate is short, which may limit the use of this complex in vivo, in cases where phase transition temperatures exceeding 35° C are required or pathologically caused temperature fluctuations at the application site are possible.

A similar pattern is observed on the rheograms of samples PEG 1500 2.5% and PEG 3400 1.5% (Figure 2e,f). For the sample, which is a binary mixture of poloxamer 407 (18.0%) and PEG 1500 (2.5%), the sol–gels phase transition based on rheological studies is fixed at a temperature of 31 °C, and the reverse gel–sol transition is fixed at 34 °C. For a composition using PEG 3400 (1.5%) as an additional gel-forming agent, a constant viscosity (0.6 ± 0.5 Pa·S) is set in the temperature range of 30–33 °C, which makes this composition the least promising of the studied pool for use in medicine. In addition, the compositions containing PEG 1500 (2.5%) and PEG 3400 (1.5%) are characterized by the lowest values of dynamic viscosity compared to those of other analyzed compositions.

On the rheogram of the sample of the binary complex of poloxamer 407 (18.0%) and PEG 1500 (1.5%) (Figure 2d), the plateau zone is fixed in the temperature range of 30–35 °C. The dynamic viscosity of the complex in the gel-like state is 1.75 ± 0.5 Pa·S, which is the best result among complexes with PEG and is comparable to the indicators of the complex with sodium alginate and the reference sample.

#### 2.1.3. Adhesive Properties of Selected Samples

**Figure 3:**
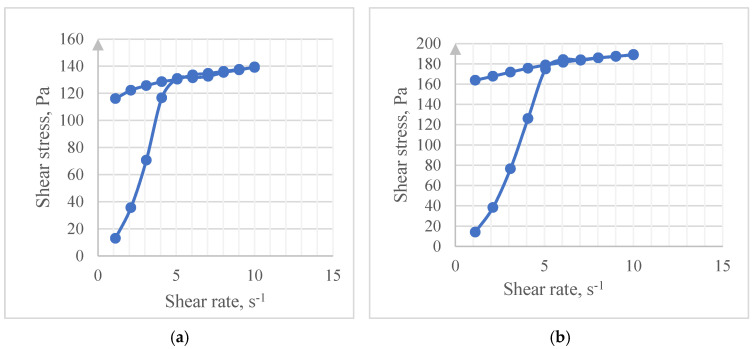

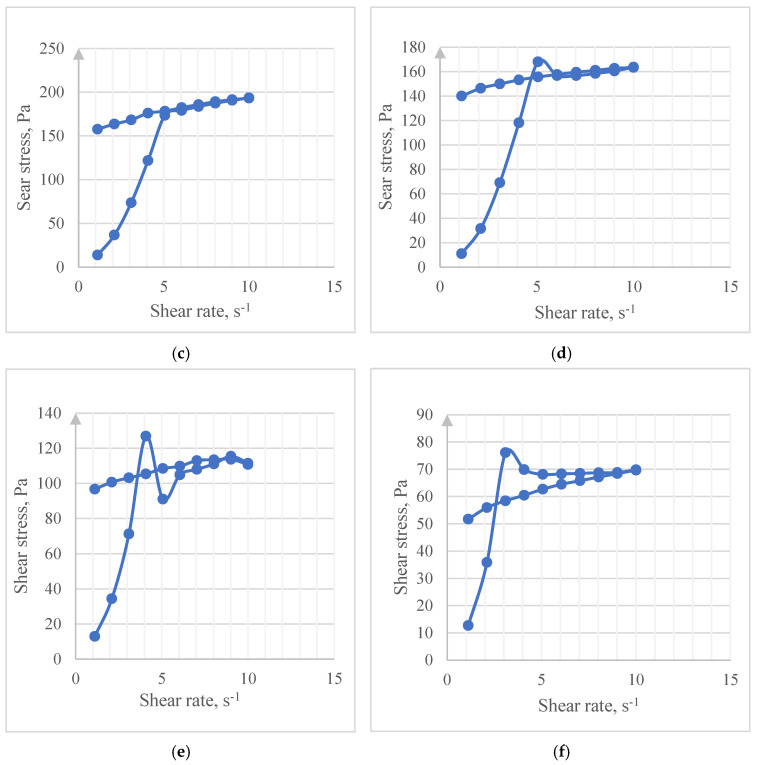
Graphs of the dependence of the shear stress on the shear rate of experimental samples, determined in the range of low speeds (from 0 to 10 s^−1^): (**a**) poloxamer 407 18.0%; (**b**) poloxamer 407 18.0%, HPMC 0.5%; (**c**) poloxamer 407 18.0%, sodium alginate 0.5%; (**d**) poloxamer 407 18.0%, PEG 1500 1.5%; (**e**) poloxamer 407 18.0%, PEG 1500 2.5%; (**f**) poloxamer 407 18.0%, PEG 3400 1.5%.

The next stage was the study of the adhesive properties of the pool of experimental samples. The measurement results are shown on rheograms (Figure 3). The measurements in this study were carried out at a variable shear rate in the small shift–large shift–small shift mode when the rotation speed of the internal geometry of the viscometer first increased sequentially, remained at the highest point for several seconds (10 s^−1^) and then gradually decreased until the rotation stopped completely. The shear stress on the above rheograms gradually increased for all analyzed samples with an increase in the shear rate from a small shift to a large shift (from 0 to 5 s^−1^), then a clearly pronounced peak of the shear stress values (Figure 3d–f) or an exit to a plateau (Figure 3a–c) was recorded. When the rotation speed decreased from a large shift to small shift (from 10 to 0 s^−1^), the shear stress remained constant or decreased slightly, without returning to the initial values.

The shear stress in this experiment recorded the presence of temporary connections between the sample and the material of the internal rotating geometry.

The suitability of this method for studying the adhesive strength of samples was determined by comparing the values of the sample viscosities and the shear stress formed in the experiment in response to the slow rotation of the geometry. Table 2 shows the average values of the dynamic viscosity of the samples at a temperature of 32 °C, which corresponds to the conditions of the experiment.

It is shown that the reference sample and the composition using sodium alginate (0.5%) have similar values of dynamic viscosity (2.3 Pa·S and 2.2 Pa·S, respectively). At the same time, the maximum values of shear stresses in the reference sample, arising in response to the rotation of the geometry of the viscometer, are in the range from 116 to 140 Pa, and those in the analyzed sample with the addition of sodium alginate were in the range from 158 to 194 Pa. Thus, the viscosity of the gel did not have a decisive influence on the magnitude and nature of the change in the shear stress determined in the experiment, and the results obtained may indirectly indicate differences in the adhesive ability of the samples.

The character of the adhesive ability of the samples was judged according to the determined range of values of shear stresses when the geometry operated in the large shift–small shift mode (Table 2). The expression of the adhesive ability of poloxamer 407 was positively affected by the addition of polymers such as HPMC, sodium alginate, and PEG 1500 (1.5%) to the mixture.

### 2.2. Discussions

To reproduce experimental data on the instability of thermosensitive solutions of poloxamer 407 obtained in the studies of Grela et al. [20], an 18.0% composition based on Kolliphor^®^ P 407 (BASF) was used as a reference sample in the experiment. The purpose of the long-term experiment was to evaluate the stability of the gelation temperature for various compositions with poloxamer 407. This was necessary because a significant variability of values has been observed over time. The ultimate goal was to ensure that the properties of the potential in situ dosage form remain unchanged during storage. 

First of all, polyethylene glycols with different molecular weights (from 1500 to 6000) were considered the introduced polymers, since their influence on the stability of compositions with poloxamer 407 was shown in studies [29]. In addition, PEGylation, the coating of nanoparticles with polyethylene glycol (PEG), is a widely employed strategy in drug and gene delivery to enhance targeting efficiency, as PEG is considered a safe and FDA-approved “stealth” polymer in the field [30]. Additionally, classical gel-forming agents (HPMC and sodium alginate) were considered auxiliary substances in concentrations preceding their critical concentration of gel formation. Hence, the three-dimensional gel structure they formed did not interfere with the reverse gel–sol transition of the composition [31,32].

It is known that poloxamer 407 is a hydrophilic nonionic surfactant, a three-block copolymer consisting of two hydrophilic blocks (from 54 to 60 polyethylene oxide units) separated by a hydrophobic block (from 95 to 105 polypropylene oxide units). Due to the presence of a hydrophilic and hydrophobic block, poloxamers have amphiphilic properties and are able to form micelles [33].

There are various hypotheses of the mechanism of micelle formation. The most plausible one is that there is an initial phase separation stage with areas that are very rich in poloxamer and areas that are more saturated with water. Then, the further process of desolvation of a part of the PPO leads to the aggregation of unimers and the formation of micelles close to each other, with the formation of clusters. As for the sol–gel transition during gel formation, the partial destruction of the PEO chains in the micellar mantle leads to the formation of a denser structure [34].

Micelles can have different shapes, namely spherical, cylindrical, or lamellar, depending on the length of the PO and EO blocks, and the core always consists of hydrophobic blocks, while hydrophilic blocks form the outer shell [35].

Thus, when polyethylene oxides with different molecular weights are added to the poloxamer solution, the micellar mantle of the poloxamer is probably strengthened, which can lead to both an improvement in the thermosensitive properties (an increase in the phase transition temperature) and a complete loss of them.

When studying mixtures of poloxamer 407 with various PEGs, the atypical behavior of gels was observed. The first feature was a strong increase in the gelation temperature compared to that of other excipients (in some cases up to 48 °C). The second feature was the long duration of gel structuring (up to 7 days, compared to 1–2 days for samples that do not contain PEG), during which the gel formation temperature dropped rapidly (up to 10–15 points), after which it reached a plateau, and the system became relatively stable.

Additionally, for some compositions containing high-molecular PEG (4000 and 6000) in high concentrations (2.0–2.5%), a very long sol–gel transition was determined. The samples remained liquid when heated in a water bath to temperatures above 50 °C; however, when placed in a thermostat in a climate chamber (constant temperature of 38 °C), a dense gel formed for several hours.

According to the experimental data obtained, the main role in the stabilization of the thermodynamic parameters of the system belongs to the molecular weight and concentration of PEG. It was noted that when using polymers with an average molecular weight (PEG 1500 and PEG 3400) at a minimum experimental concentration (1.5%), it was possible to stabilize the thermosensitive system for the entire period of the study (12 weeks).

During the conduction of the long-term studies, the following phenomenon was identified. When PEG 1500 was added to the poloxamer 407 at a concentration of 2.0%, in contrast to the introduction of PEG 3400 in a similar concentration, the system was not stabilized until the fifth week of the experiment, and the gelation temperature of the composition was kept at 23.5 °C, decreasing after this to 20 °C and becoming stable. The thermal reversibility of the composition was preserved; however, the low temperature of the sol–gel transition made the use of the composition unpromising.

It should be noted that the composition with PEG 1500 in a concentration of 2.5% demonstrated excellent stability and a high average gelation temperature (about 29 °C). This phenomenon requires further detailed study using modern analytical methods. The use of sodium alginate gelling agents and HPMC as stabilizers of the thermoreversible system at a concentration of 0.5% was considered highly promising since it provided a relatively high average temperature of gelling (29 °C and 27.8 °C, respectively), which remained stable throughout the entire period of the experiment. The gelation times for these compositions were also average among those for the entire analyzed pool.

The rheological methods of studying the indicators of thermosensitive in situ systems used in this study also showed their prospects. Rotary viscometers with a temperature-controlled system are examples of affordable laboratory and analytical equipment, and the methods produced with their help are highly reproducible and can be validated. The temperature test for analytical samples at a constant shear rate made it possible to conduct a reasonable and fast screening of compositions based on the indicator of the duration of the plateau zone—a high dynamic viscosity with increasing temperature. The results reflected in the rheograms (Figure 2) are visual and can be used for the effective selection of promising samples.

The proposed rheological test for studying the adhesion of samples gives an indirect forecast of the degree of adhesion of a composition. However, as it was noted earlier, it is easily reproducible and available in most laboratories, unlike the well-known mucoadhesive screenings, and can be used as an additional factor in choosing the optimal composition, along with the main rheological test. 

Future advances in this area may be supported by linking nanostructural characterization techniques to spectroscopic tools to probe into the interplay between changes in structure and inter-macromolecular interactions and provide a rational basis for additive selection [36].

## 3. Conclusions

In the course of the conducted studies, the effect of the introduced additional polymers on the stability of the thermosensitive compositions of poloxamer 407 was shown. The best results of stability studied during 12 weeks of storage (at a temperature of +5/+8 °C), demonstrated the following compositions: poloxamer 407 18.0%, HPMC 0.5%; poloxamer 407 18.0%, sodium alginate 0.5%; poloxamer 407 18.0%, PEG 1500 1.5%; poloxamer 407 18.0%, PEG 1500 2.5%; poloxamer 407 18.0%, PEG 3400 1.5%; poloxamer 407 18.0%, PEG 3400 2.0%. Thus, the positive effect of the addition of medium-molecular-weight PEGs in the minimum operating concentrations (1.5–2.0%), as well as that of gel-forming agents (HPMC, sodium alginate) in a concentration lower than the critical concentration of gel formation (0.5%) was shown.

According to the data obtained after rheological tests, three experimental binary systems based on poloxamer 407 (18.0%) can be distinguished as the most promising for creating targeted drug delivery systems based on them, according to the parameters of gelation temperature, gelation time, and stability, as well as mucoadhesion with the addition of HPMC 0.5%, sodium alginate 0.5% and PEG 1500 1.5%.

Poloxamer 407 is a thermoreversible gel with a low viscosity at room temperature that rapidly transforms into a viscous gel at body temperature (approximately 37 °C). It can be conveniently administered using a syringe or applicator, facilitating in situ gelation at the desired location [36]. Additionally, polymer micelles have proven to be effective carriers for compounds with poor solubility, unfavorable pharmacokinetics, and low stability in the physiological environment [37]. These promising formulations, exhibiting improved pharmaceutical properties, can be selected for further development as dosage forms with active substances. Moreover, the gelation temperature can be adjusted to meet the specific requirements of the study, ranging from room temperature to body surface temperature, and even to pathological temperatures associated with inflammation.

Studies of the issues of stabilization of specific indicators of thermosensitive systems will be continued in the future using other analytical methods as well as by creating non-binary systems.

## 4. Materials and Methods

To develop thermoreversible compositions, the poloxamer 407 Kolliphor^®^ P 407 (BASF SE, Ludwigshafen, Germany) was used in the experiment, as well as the following excipients for the formation of stable polycomplexes: PEG 1500-Pluriol^®^ E 1500; PEG 3400-Pluriol^®^ E 3400; PEG 4000-Pluriol^®^ E 4000; PEG 6000-Pluriol^®^ E 6000 (BASF SE, Ludwigshafen, Germany); hydroxypropylmethylcellulose Benecel^®^ K100M PHARM; Sodium Alginate Protanal^®^ CR 8133 (FMC, Philadelphia, PA, USA). Gelling systems were prepared using ‘Cold Methods’. All compositions were binary mixtures of poloxamer 407 and an additional gel-forming agent. The concentration of poloxamer 407 in all experimental samples was constant and amounted to 18.0%. The polymer concentrations ranged from 0.5 to 2.5% depending on weight. The weighed sample of the poloxamer was dispersed in purified water, mixed with an additional polymer of a given weight, and cooled to −15/−18 °C for 5–7 min until it was completely dissolved.

### 4.1. Long-Term Screening Test

Temperature and gelation time in a long-term experiment, as well as viscosity using a rotational temperature test and adhesion using the rotational viscosity test, were determined and proposed as new screening parameters for evaluating thermoreversible systems.

The gelation temperature was measured in the samples at a volume of 20 mL +/−0.5 mL that were stored for 12 weeks of the experiment in a refrigerator at a temperature of +5/+8 °C, by heating them in a water bath at a constant temperature of 50 °C with an immersion measuring thermosonde with a periodicity of 7 days.

The gelation time was measured from the moment the sample was immersed in a polymer container in a water bath at 50 ± 1 °C. The values determined by increasing the dynamic viscosity of the sample during its mixing were taken as an indicator of the gelation temperature and time.

To process the obtained data, statistical methods were used to calculate the average value (gelation temperature) and the variance of a random variable, which was taken as a measure of the stability of the samples. 

### 4.2. Rheological Property Test

The MS-DIN 33 measuring system (Lamy, Rhône-Alpes, France) was utilized for the test. It consists of an internal rotating cylinder with a cone-shaped tip and an internal static cylinder. The recommended sample loading volume is approximately 17 mL.

For the rheological tests assessing the thermal sensitivity of the developed systems, the dependency of dynamic viscosity (Pa·S) at a constant shear rate of 100 s^−1^ on temperature was determined. The measuring geometry of the device was placed in THERMACELL PACKAGE RT-1 Plus (La, Paris, France). The sample temperature gradually increased from 20 to 50 °C during the test, and the measurements were carried out in three repetitions with an interval of 5 min.

### 4.3. Adhesion Property Test

The adhesion value was measured using the rheological method in the range of shear rates from 0 to 10 s^−1^ at a constant temperature of 32 ± 0.5 °C, which simulates the conditions of external and topical application of the system by the patient. The nature and magnitude of adhesion were judged by the shape of the rheological curve, as well as by the maximum values of the shear stress that occurred during the experiment.

### 4.4. Statistical Analysis

In the statistical study, Microsoft Excel 2018 was utilized for data analysis. The formulas employed included standard deviation (STDEV) and sample variance (VAR). The STDEV formula was applied to calculate the standard deviation for a sample of a population, while the VAR formula was used to determine the sample variance. The level of statistical significance was defined at *p* < 0.05.

## Figures and Tables

**Table 1:** Average temperature measurement results of gelation time for experimental samples.

Polymer Excipients n = 3	The Average Value of the Gelation Temperature, °C	Standard Deviation	Sample Variance	Internal Scatter
Reference composition	26.68	1.832	3.358	[22.0; 29.0]
PEG 1500 1.5%	29.85	1.699	2.888	[27.5; 33.0]
PEG 1500 2.0%	27.05	9.923	98.475	[20.0; 47.0]
PEG 1500 2.5%	28.92	1.294	1.674	[27.0; 31.5]
PEG 3400 1.5%	30.13	1.400	1.960	[28.0; 33.0]
PEG 3400 2.0%	28.64	1.036	1.074	[27.0; 31.0]
PEG 3400 2.5%	30.62	4.160	17.307	[27.0; 42.0]
PEG 4000 1.5%	29.80	8.098	65.575	[20.0; 42.0]
PEG 4000 2.0%	27.74	11.055	122.218	[20.0; 48.0]
PEG 4000 2.5%	33.72	7.380	54.470	[28.0; 50.0]
PEG 6000 1.5%	29.05	6.792	46.135	[22.0; 42.0]
Sodium alginate 0.5%	29.09	1.241	1.541	[27.0; 31.0]
HPMC 0.5%	28.50	1.780	3.167	[26.0; 31.5]

**Table 2:** Results of rheological measurements of experimental samples.

Composition of the Analyzed Sample n = 3	Average Dynamic Viscosity (100 s^−1^) at a Temperature of 32 °C	Standard Deviation	Sample Variance	Ranges of Average Values of the Shear Stress in the Large Shift–Small Shift Mode (from 10 to 0 s^−1^)
Reference sample	2.3 Pa∙s	0.187	0.035	116–140 Pa
HPMC 0.5%	2.6 Pa∙s	0.107	0.011	164–189 Pa
Sodium alginate 0.5%	2.2 Pa∙s	0.135	0.018	158–194 Pa
PEG 1500 1.5%	1.8 Pa∙s	0.164	0.027	140–164 Pa
PEG 1500 2.5%	1.2 Pa∙s	0.072	0.005	97–114 Pa
PEG 3400 1.5%	0.6 Pa∙s	0.207	0.043	52–70 Pa

## Data Availability

The authors can provide any necessary information on the study upon request.
